# The effects of a medical hypnotherapy on clothing industry employees suffering from chronic pain

**DOI:** 10.1186/1745-6673-8-25

**Published:** 2013-09-25

**Authors:** Zenija Roja, Valdis Kalkis, Inara Roja, Henrijs Kalkis

**Affiliations:** 1 Ergonomics Research Centre, University of Latvia, Riga, Latvia; 2 Out-patient Department of the Riga 1st Hospital, Riga, Latvia; 3 Faculty of Economics and Management, University of Latvia, Riga, Latvia

**Keywords:** Muscle fatigue, Work heaviness, Psychotherapeutic intervention, Quality of life

## Abstract

**Background:**

Problems associated with pain in several body regions due to work-related musculoskeletal disorders (WRMDs), repetitive movement and negative stress at work are quite common in many manufacturing industries of Latvia, int.al. clothing industry. The aim of this study was to evaluate efficiency of the psychotherapeutic intervention using medical hypnotherapy (MH) program for mind-body relaxation with pain-blocking imagery, cognitive restructuring of unpleasant physical and emotional experience.

**Methods:**

300 sewers and 50 cutters with chronic pain were involved in the study. Self-rated WRMDs symptoms, pain intensity and interference were assessed using the extended version of Nordic Musculoskeletal Questionnaire and Brief Pain Inventory Scale. Assessment of the functional state of muscles was carried out using myotonometric (MYO) measurements. Work heaviness degree was estimated via heart rate monitoring (HRM). The MH program was composed of cognitive hypnotherapy and self-hypnosis training. Sunnen Trance Scale was used to determine person’s hypnotic susceptibility. Life quality assessment before and after MH program was carried out using Quality of Life Scale.

**Results:**

At the beginning of MH program sessions both sewers and cutters reported on pain interference with general activities, mood, sleep, normal work, etc., but after MH the interference of pain significantly decreased. HRM data confirmed that work heaviness degree of sewers and cutters can be referred to as light and moderate work (energy expenditure for their tasks varies from 3.4 till 4.7 kcal/min). Using MYO measurements it was stated that before MH 22% of workers involved in the study fell under III MYO category indices, consequently, their muscle tone was increased, which is associated with muscular fatigue. After MH muscle tone remained within the normal range meaning that they were able to adapt to the existing workload (II MYO category) or fully relax (I MYO category).

**Conclusions:**

MH program including exercises-workouts, cognitive hypnotherapy and self-hypnosis training sessions is an effective method to decrease composite chronic pain intensity for sewers and cutters, as well as to decrease psychogenic tension and muscle fatigue (proved by objective measurements of muscles tone) and to increase the life quality.

## Background

WRMDs and muscle pain affecting the wrists, shoulders, neck and back are common problems for workers in different industries, including clothing industry, and are caused by ergonomic risks [1,2]. The risks for clothing industry employees’ - sewing machine operator’s (sewers), cutters, etc., in many cases have been linked with poor workstation design, and organizational factors such as the piecework system. Work monotony and compulsory postures promote fatigue and other work-related health problems. Therefore, the respective muscle group tension is considered to be the main cause of pain. A high occurrence of musculoskeletal complaints among clothing industry employees has been already described by many authors [3-8]. It is known that high work pace, lack of control over work, and insufficient co-workers’ support can lead to muscles fatigue and chronic pain in different body parts [9,10]. At the same time it is estimated that 30–54% of chronic pain patients suffer from severe forms of depression caused by stress at work [11]. These persons are less active and report on greater disability and interference with their daily activities due to pain [12,13]. It has serious social, psychological and economical consequences in Latvia, because it is often associated with long-term employee absenteeism due to illness or even disability, which to a certain extent influences workers’ life quality in general.

The psychotherapeutic intervention included in various rehabilitation (returning to work) program are widely used to help employees to make positive changes as regards their working life [14,15]. It is established that intensive physical training (workouts for the whole body) and psychosocial support for patients with chronic pain significantly improved some aspects of health-related quality of life in addition to pain relief and renewal of functions [16-18].

Literature sources reveal that majority of studies have focused this far only on sewing machine operators, leaving workers in other jobs out. Therefore, the aim of this research was to determine the effectiveness of the MH for clothing industry employees suffering from chronic pain in different bodily parts, focusing mainly on the neck/shoulders, hand, arms and legs. This study consists of three parts:

1. A cross-sectional survey of all participants having completed questionnaires, thus providing us with information regarding the body part with chronic pain;

2. A survey of the persons who had agreed to take part in MH program (completed questionnaires on intensity of pain, psychosocial work environment conditions, hypnotic susceptibility, and life quality);

3. Objective investigation - heart rate monitoring (assessment of work-related energy expenditure), and myotonometric measurements to determine the muscle fatigue of selected persons.

## Methods

### Study population

Sewers and cutters from various biggest clothing enterprises and garment factories in Latvia were chosen. Workers, who were included, had to have a more or less fixed workplace and had to be able to understand and fill out questionnaires. 350 persons (300 sewers and 50 cutters) were involved in the research. Participants were divided into three groups according to the length of their employment in this job: group I, 0-7 years (n = 180); group II, 8-15 years (n = 102), and group III, more than 15 years (n = 68). Participants completed questionnaires, providing us with the information regarding the body part that suffered from pain, duration of pain, length of service in the profession, age, education, as well as their physical activities and other activities. The study was carried out according to the Helsinki Declaration, the local ethics committee approved the study design, and all participants gave their consent. Background factors are shown in Table 1.

**Table 1:** Background factors of the subjects, length of service, mean age, and range

**Population (length of service)**	**n**	**Mean age ±SD**	**Range**
Sewers	300	37.3±10.5	18-65
(0–7 years)	150	25.2±6.3	18-35
(8–15 years)	90	35.9±5.1	26-42
(> 15 years)	60	53.6±7.7	46-65
Cutters	50	35.9±10.5	22-65
(0–7 years)	30	32.8±6.1	22-40
(8–15 years)	12	40.4±6.8	30-48
(> 15 years)	8	56.9±7.6	42-65

Our research was approved by the Human Ethics and Institutional Review Board of Riga Stradins University.

### Participants in the MH program

Persons who participated in the MH program were selected as follows. The MH program was proposed to persons (n = 210) with chronic neck, shoulders, arms, and hands (NSAH) pain lasting for 3–4 month or more, and who have had a negative previous experience with medication therapy. 60 sewers (50 female and 10 male) and 30 cutters (20 female and 10 male) of 210 employees agreed to take part in the MH program including objective investigation (HRM, MYO) and all of them successfully fulfilled the MH program. They preferred to use mind-body relaxation training, to acquire self-hypnosis with the aim to increase personal control over chronic pain at work. The inclusion criteria were: full consent to participate in the study, age and length of service; presence of chronic NSAH pain, also in the back and legs (medical examination data); performance of relaxation exercises during the work breaks, and remedial gymnastics or swimming at least twice per week. The exclusion criteria were: acute pain in the different bodily regions; inflammatory rheumatic disease and disorders caused by trauma, having not visited doctor for mandatory medical examinations. The control group was not formed. Some employees categorically refused to take part in MH program, as well as to undergo performing of objective measurements (HRM, MYO). The examination, including MH program, was carried out in a one year period. Background factors of the reference groups are shown in Table 2.

**Table 2:** Background factors of the reference groups, length of service, mean age and range, mean height, mean weight, mean body mass index (BMI), and mean rest heart rate (RHR)

**Population (length of service)**	**n**	**Mean age ± SD**	**Range**	**Mean height, cm ± SD**	**Mean weight, kg ± SD**	**Mean BMI, kg/m**^**2 **^**± SD**	**Mean RHR, beats/min ± SD**
Sewers	60	41.8±18.6	18-65	164.2±11.7	77.2±13.7	29.3±2.0	75.5±7.3
(0–7 years)	20	22.7±5.2	18-35	159.3±10.7	73.1±12.1	26.1±1.9	59.7±9.6
(8–15 years)	30	33.8±5.2	26-42	165.5±12.6	74.8±11.1	26.7±1.7	73.3±8.2
(> 15 years)	10	52.8±8.9	46-65	162.1± 0.5	77.5±10.0	29.1±3.5	76.0±8.6
Cutters	30	41.2±19.2	22-65	167.5±18.7	71.4±10.2	26.4±4.1	70.7±7.6
(0–7 years)	12	27.8±6.6	22-40	170.6±14.8	69.5±9.8	25.8±3.3	55.7±8.8
(8–15 years)	10	30.6±6.4	30-48	168.6±12.4	71.5±9.7	26.7±3.7	59.7±8.7
(> 15 years)	8	56.9±7.6	42-65	166.6±11.9	73.5±11.3	27.2±3.8	63.7±7.8

### Data collection

#### Extended version of Nordic musculoskeletal questionnaire (NMQ-E)

**Figure 1:**
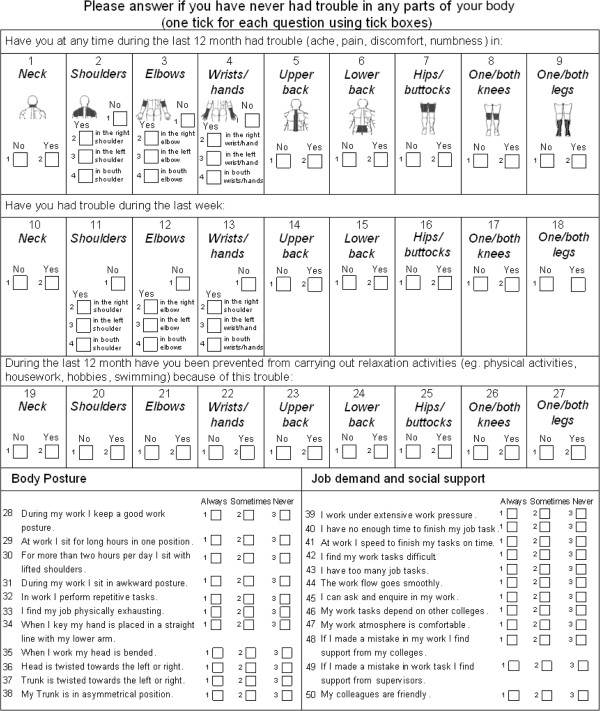
Musculoskeletal questionnaire (trouble with the locomotive organs and work disposition).

In the research the modified (extended) version of Nordic musculoskeletal questionnaire was used to assess musculoskeletal problems of sewers and cutters (the nature and severity of self-rated musculoskeletal symptoms, including items inquiring about the experience of problems in nine body areas) [19]. In our study, the extended version of NMQ-E contains some additional questions regarding body postures; job demands and social support (see Figure 1).

#### Brief pain inventory (BPI)

The BPI was used to provide information on pain intensity (the sensory dimension), as well as on the degree, to which pain interferes with function (the reactive dimension) [20]. The BPI includes questions (in total 9) regarding pain relief, pain quality, patient’s perception of the cause of pain and how pain has interfered during the past 24 hours with general activity, mood, walking ability, normal work, relations with other people, sleep, and enjoyment of life. BPI provides a continuous scale for subjective magnitude estimation of the symptom to be evaluated: 0 = "no pain"; 0–2 "mild"; 3–5 "moderate"; 6–8 "severe"; 9–10 "worst disability". The composite pain intensity and composite pain interference were calculated. The BPI was also used to assess the effectiveness of MH program.

#### Sunnen trance scale (STS)

STS is a scoring form for hypnosis, self- hypnosis, and meditation [21]. In this study, STS was used as a method to determine one’s hypnotic susceptibility, as well as abilities to further self-hypnosis or meditation. The scale is composed of 20 subscales, each graded from 0 to 5. The goal is not to reach 100 in total.

#### Quality of life scale (QOLS)

Life quality assessment was carried out before and after MH program, using QOLS, offered by American Chronic Pain Association [22]. QOLS is aimed to help people, suffering from pain, and their health care team to evaluate and communicate the impact of pain on the basic activities of their daily life. With this scale, patients rate their functioning to determine how much their pain is disrupting their quality of life. The range of this scale is from 0 (someone who is non-functioning, stays in bed all day, and feels helpless about life) to 10 (someone who has a normal quality of life: goes to work each day, does daily activities, and takes an active part in family life and social life outside the work).

### Objective investigations

#### Myotonometry (MYO)

Assessment of the functional state of skeletal muscles and muscle fatigue was carried out using myotonometric measurements performed with MYOTON-3 device created in Estonia, the University of Tartu. Theoretical concept of MYO is described in references [23,24], as well as in our earlier publication describing results of the research on skeletal muscle fatigue of road maintenance workers[25]. The working principle of MYO lies in using acceleration probe to record how peripheral skeletal muscle or its part reaction to the mechanical impact (testing end mass 20 grams, duration 15 milliseconds) and the following analysis of the resulting signal. The frequency of the damped oscillations (Hz), measured during the rest period, characterizes the tissue tone. Muscles stiffness (N/m) reflects the resistance of tissue to the force that changes its shape. Calculations are done using measuring device and considering the relationship: *m* · a_max_/*Δl*, where *m* is the mass of the testing end of device; *a*_*max*_ is the maximal amplitude of oscillation, and *Δl* is the depth of the displacement of the testing end. The procedure of NSAH muscle testing was performed in a sitting position, the muscle length was middle; for all measurements the subject took the same position. Thus, it is possible to obtain the most precise results, when estimating muscle fatigue or the ability to restore elastic muscle qualities after the work cycle.

#### Heart rate monitoring (HRM)

The work heaviness degree considering workers physical activity (intensity) was estimated by HRM. Measurements were based on heart rate variation, which correlates with oxygen consumption and allows quantifying the objective energy expenditure for each work phase including short rest periods [26]. HRM was performed using *POLAR S810i*™ Heart Rate Monitor device with data processing software. The device sums up the acquired heart rate data and transforms them into metabolic energy consumption - E, kcal/min. Work heaviness in terms of energy expenditure was classified according to NIOSH standard [27] and ISO 28996 (I - light work, II - moderate, III - hard, IV - very hard, V - ultimate work). For example, light work is classified as E = 2.0-4.9 kcal/min for a male and 1.5-3.4 kcal/min for a female; very hard work - 10.0-12.4 and 7.5-9.4 kcal/min respectively.

### The MH program

The MH program was continued for 9 months and included cognitive hypnotherapy (CH) [28] and self-hypnosis (SH) [29]. At the same time all participants continued performing relaxation exercises during the work breaks (2–3 minutes after 60 minutes of work), and remedial gymnastics or swimming (45 minutes twice per week) after the work. T hese activities are aimed to reduce emotional tension, as well as to promote the relaxation of tired muscles.

The relaxation exercises during the work-brakes consisted of various energetic practices (in sitting and standing positions), in which extensor and flexor muscles are regularly stretched, tensed and then relaxed (1 minute). The rest part of the break (1–2 minutes) was used for passive relaxation when participants were asked to close their eyes or look out of the window into the distance: breathe deeply and rhythmically, relax the muscles. These exercises were mastered under physiotherapist’s guidance and then performed independently during the breaks.

The remedial gymnastics after the work applying special exercises prescribed by a doctor was carried out (10 persons) in a group supervised by the physiotherapist. Swimming was done in swimming pool (10 persons in a group, as well) supervised by the instructor. Before starting activities the instructor mentored all persons for warm-up techniques, proper breathing, and muscle relaxation during the swimming.

CH and SH were used as a therapy/rehabilitation method. CH is a highly structured and systematic MH modality consisting of several phases: *intro phase* (I) – *trance phase* (II) *– hypnodrama phase* (III) – *final phase* (IV) – *exit-from-trance phase* (V). During CH sessions involving deep mind-body relaxation, pain-blocking imagery, cognitive restructuring of unpleasant physical and emotional experience, individuals learn to master self-hypnosis and to monitor their pain. In the beginning of MH program the CH and SH group treatment sessions were carried out. These sessions lasted for 3 month and consisted of twelve 60-minute CH and SH sessions alternately every week supervised by certified physician - hypnotherapist. During SH sessions each participant of the group was trained in self-hypnosis and asked to practice self-hypnosis individually every day for 10-15 minutes, using a CD with self-hypnosis induction and suggestions. All patients were trained, how to complete the pain diary, and were asked to make records on their pain and other feelings in the pain diary in the beginning, during and in the end of the MH program. The beneficial effects of hypnosis were determined after the last CH and SH group treatment session and in follow-ups (3- and 6- months) by contacting the patients viae-mail and asking to complete and return a post-treatment assessment that included the BPI items. BPI scores were summed up to provide data on composite pain intensity (sensory dimension) and composite pain interference (reactive dimension).

### Statistical analysis

The acquired results were processed, using statistical data processing software SPSS.16 (SPSS Inc., Chicago, IL) according to popular descriptive statistical methods. Confidence interval (95% CI) and prevalence proportion ratio (PPR) were used to indicate the reliability [30]. Reliability interval (inter-rater agreement) was also calculated and Cohen’s Kappa (κ) coefficient was determined [31]. This coefficient identifies connectivity of the experimental data, the number of participants and the proportion or correlation of the participants’ acceptance of the experimental data: κ = (P_O_ - P_C_)/(1 - P_C_), where: P_O_ – correspondence proportion of objective experimental data with respondents’ responses (yes or no), P_C_ - correspondence proportion of data with number of participants (P_C_ = Σp_i_^2^, where p_i_ is acceptance of each participant, expressed in percent or as fractional number).

## Results

### Multivariate analyses of the sewers and cutters with chronic pain

The distribution of persistent complaints in each part of the body, separately for sewers and cutters according to the extended version of Nordic musculoskeletal questionnaire NMQ-E was shown in the Tables 3 and 4. The distribution of postures, job demands and social support was shown in the Tables 5 and 6. For the reference group, length of service as a sewing- or cutter machine operator was used as a measure of exposure, including time spent in the relevant factory and time of work in other garment factories.

**Table 3:** **Distribution of persistent complaints in different parts of the body of *sewers*, prevalence proportion ratio (PPR) and 95% confidence interval (CI), compared with joint group (n = 300)**

	Sewers* (n = 300)		**Reference groups (length of service, years)**					
			**I (0–7) (n = 150)**		**II (8–15) (n = 90)**		**III (> 15) (n = 60)**	
	**Number**	**%**	**Number**	**%**	**Number**	**%**	**Number**	**%**
			**PPR**	**(95% CI)**	**PPR**	**(95% CI)**	**PPR**	**(95% CI)**
Neck	107	35.7	24	16.0	72	80.0	11	18.3
			0.45	(0.38-0.52)	2.24	(1.78-2.70)	0.51	(0.40-0.61)
Shoulder	130	43.3	46	30.6	67	74.4	17	28.3
			0.71	(0.59-0.82)	1.72	(1.36-2.07)	0.65	(0.51-0.78)
Elbow	28	9.3	4	2.6	14	15.5	10	16.6
			0.28	(0.23-0.32)	1.66	(1.31-2.00)	1.78	(1.41-2.15)
Wrist/hands	65	21.6	11	7.3	39	43.3	15	25.0
			0.34	(0.28-0.39)	1.99	(1.57-2.40)	2.98	(2.36-3.59)
Upper back	35	11.7	0	0	27	30.0	8	13.3
			0		2.57	(2.04-3.10)	2.14	(1.70-2.58)
Low back	17	5.7	1	0.6	10	11.1	6	10.0
			0.12	(0.10-0.14)	1.95	(1.54-2.35)	2.35	(1.86-2.83)
Hip/Thigh	10	3.3	0	0	3	3.3	7	11.6
			0		1.00	(0.79-1.20)	3.00	(2.38-3.62)
Knee	5	1.6	0	0	2	2.2	3	5.0
			0		1.33	(1.05-1.60)	3.00	(2.38-3.62)
Ankle/Shank/Feet	7	2.3	2	1.3	2	2.2	3	5.0
			0.57	(0.48-0.66)	0.95	(0.75-1.14)	2.14	(1.70-2.58)

*Multiple answers were possible.

**Table 4:** **Distribution of persistent complaints in different parts of the body of *cutters*, prevalence proportion ratio (PPR) and 95% confidence interval (CI), compared with joint group (n = 50)**

	Cutters* (n = 50)		**Reference groups (length of service, years)**					
			**I (0–7) (n = 30)**		**II (8–15) (n = 12)**		**III (> 15) (n = 8)**	
	**Number**	**%**	**Number**	**%**	**Number**	**%**	**Number**	**%**
			**PPR**	**(95% CI)**	**PPR**	**(95% CI)**	**PPR**	**(95% CI)**
Neck	27	54.0	9	30.0	10	83.3	8	100
			0.55	(0.35-0.75)	1.54	(0.67-2.41)	1.85	(0.56-3.13)
Shoulder	36	72.0	18	60.0	11	91.6	7	87.5
			0.84	(0.54-1.14)	1.27	(0.55-1.98)	1,22	(0.37-2.06)
Elbow	15	30.0	4	13.3	8	66.6	3	33.3
			0.44	(0.28-0.59)	2.22	(0.96-3.47)	1.25	(0.38-2.11)
Wrist/hands	24	48.0	6	20.0	10	83.3	8	100
			0.42	(0.27-0.57)	1.73	(0.75-2.71)	2.08	(0.64-3.52)
Upper back	8	16.0	0	0	5	41.6	3	37.5
			0		2.60	(1.13-4.07)	2.35	(0.72-3.98)
Low back	7	14.0	0	0	2	16.6	5	62.5
			0		1.19	(0.51-1.86)	4.46	(1.37-7.55)
Hip/Thigh	0	0	0	0	0	0	0	0
			0		0		0	
Knee	13	26.0	1	3.3	6	50.0	7	87.5
			0.13	(0.08-0.17)	1.92	(0.83-3.00)	3.38	(1.03-5.72)
Ankle/Shank/Feet	22	44.1	11	36.6	9	75.0	2	25.0
			1.00	(0.64-1.36)	2.05	(0.89-3.20)	0.68	(0.21-1.15)

*Multiple answers were possible.

**Table 5:** **Distribution of postures, job demand and social support for *sewers*, prevalence proportion ratio (PPR) and 95% confidence interval (CI), compared with joint group (n = 300)**

	Sewers* (n = 300)		**Reference groups (length of service, years)**					
			**I (0–7) (n = 150)**		**II (8–15) (n = 90)**		**III (> 15) (n = 60)**	
	**Number**	**%**	**Number**	**%**	**Number**	**%**	**Number**	**%**
			**PPR**	**(95% CI)**	**PPR**	**(95% CI)**	**PPR**	**(95% CI)**
Awkward posture	275	91.6	141	94.0	79	87.7	55	91.6
			1.02	(0.85-1.18)	0.96	(0.76-1.15)	1.00	(0.74-1.25)
Physical exhausting	80	26.6	14	9.3	51	56.6	15	25,0
			0.35	(0.29-0.41)	2.13	(1.69-2.57)	0.94	(0.70-1.17)
Extensivework	20	6.6	8	5.3	7	7.7	5	1.6
			0.80	(0.67-0.92)	1.67	(1.32-2.01)	1.25	(0.93-1.56)
High work speed	222	74.0	110	73.3	72	80.0	40	66.6
			1.00	(0.84-1.16)	1.08	(0.85-1.30)	0.90	(0.67-1.13)
Too manyjob tasks	148	49.3	45	30.0	68	75.5	35	58.3
			0.61	(0.51-0.70)	1.54	(1.22-1.85)	1.19	(0.89-1.49)
Colleagues’ support	268	89.3	142	94.6	82	91.1	44	73.3
			1.07	(0.89-1.24)	1.03	(0.82-1.24)	0.82	(0.61-1.03)
Supervisors’ support	242	80.6	130	86.6	76	84.4	36	60.0
			1.08	(0.90-1.25)	1.05	(0.83-1.26)	0.75	(0.56-0.94)

*Multiple answers were possible.

**Table 6:** **Distribution of postures, job demand and social support for *cutters*, prevalence proportion ratio (PPR) and 95% confidence interval (CI), compared with joint group (n = 50)**

	Cutters* (n = 50)		**Reference groups (length of service, years)**					
			**I (0–7) (n = 30)**		**II (8–15) (n = 12)**		**III (> 15) (n = 8)**	
	**Number**	**%**	**Number**	**%**	**Number**	**%**	**Number**	**%**
			**PPR**	**(95% CI)**	**PPR**	**(95% CI)**	**PPR**	**(95% CI)**
Awkward posture	47	94.0	28	93.3	11	91.6	8	100
			0.99	(0.63-1.34)	0.97	(0.42-1.51)	1.06	(0.32-1.79)
Physical exhausting	38	76.0	21	70.0	10	83.3	7	87.5
			0.91	(0.58-1.23)	1.09	(0.47-1.70)	1.15	(0.35-1.94)
Extensivework	45	90.0	27	90.0	10	83.3	8	100
			1.00	(0.64-1.35)	0.92	(0.40-1.44)	1.11	(0.34-1.88)
High work speed	20	40.0	12	40.0	4	33.3	4	50.0
			1.00	(0.64-1.35)	0.83	(0.36-1.29)	1.25	(0.38-2.12)
Too manyjob tasks	41	82.0	27	90.0	9	75.0	5	62.5
			1.10	(0.70-1.49)	0.92	(0.40-1.44)	0.76	(0.23-1.28)
Colleagues’ support	38	76.0	27	90.0	5	41.6	6	75.0
			1.18	(0.75-1.60)	0.54	(0.23-0.84)	0.98	(0.30-1.66)
Supervisors’ support	45	90.0	24	80.0	12	100	7	87.5
			0.89	(0.57-1.21)	1.11	(0.48-1.73)	0.97	(0.29-1.64)

*Multiple answers were possible.

Results displayed in Table 3 show that 35.7% of sewers generally complain about pain in their neck region, 43.3% - in shoulders, 21.6% - in palm and 11.7% - in upper part of the back. It should be noted that the majority of complaints regarding the pain in the mentioned bodily parts were uttered by sewers aged 26–42 with the length of service 8-15 years (PPR = 1.99-2.24; CI = 1.57-2.70): 80.0% - neck region, 74.4% - shoulders, 43.3% - palm un 30.0% - upper part of the back.

Statistical data presented in Table 4 show that 54.0% of cutters complain about pain in their neck region, 72.0% - shoulders, 48.0% - palm and 44.0% - shins. Further analysis of the acquired data reveals that majority of cutters (87-100%) aged 42 – 65, with the length of service above 15 years, report on pain or discomfort in their neck, shoulders and knees (PPR = 1.27-3.38; CI = 0.37-3.20). Cutters aged 30-48 with the length of service from 8 to 15 years slightly less complain of having pain in the mentioned bodily parts (PPR = 1.22-2.05; CI = 0.55-5.72). Furthermore, 75.0% reported also about pain in their shins (PPR = 2.05; CI = 0.89-3.20).

Statistical data displayed in Table 5 clearly show that almost all sewers have to work in forced awkward postures and at a great speed. Quite many sewers recognize being supported by their colleges and supervisors; it especially refers to younger employees with the length of service under 7 years (94.6% and 86.6%) and middle-aged sewers having worked for 8 to 15 years (91.1% and 84.4%). In addition, young people aged 18 to 35 years with the length of service less than 7 years do not consider work intensity to be an important factor; only 5.3% have mentioned it in their inquiries.

Statistical data in Table 6 show that cutters’ work means working in awkward forced postures (94.0-100%), their work is varied (90.0-100%), and the greatest support is received from their supervisor (87.5-90.0%). It must be added that younger cutters, with the length of service less than 7 years, believe that they have too much tasks (90.0%), when compared to others, whose length of service is longer.

### Analysis of the MH program results

#### CH and SH treatment

According to the MH program design, CH and SH group treatment sessions alternately every week were performed within 3 months, followed by a 6-month program of self-hypnosis practice. It is noted that remedial gymnastic exercises were continued throughout the MH program.

Before CH and SH treatment the hypnotic susceptibility using STS was performed. The results of hypnotic susceptibility of the examined persons (sewers, n = 60 and cutters, n = 30) before the MH program are shown in Table 7.

**Table 7:** **Comparison of hypnotic susceptibility of *sewers *(n = 60) and *cutters *(n = 30) according Sunnen trance scale at the beginning of MH program (before cognitive hypnotherapy session was started), mean scores, 95% confidence interval (CI), and P**_**0 **_**- proportion of observed agreement**

**Items**	**Reference group**	**Before MH program**			
		**Number**	Mean scores*±SD	**CI**	**P**_**0**_
0 - Immunity	Sewers	6	15.9±3.7	11.8-19.9	0.96
	Cutters	5	20.0±5.9	12.8-27.1	0.89
I - Light trance	Sewers	34	33.0±8.8	24.6-41.3	0.88
	Cutters	12	40.3±8.3	25.8-54.3	0.78
II - Medium trance	Sewers	10	63.4±7.3	47.3-79.4	0.95
	Cutters	9	57.8±8.6	42.6-71.4	0.96
III- Distinct trance	Sewers	8	75.7±5.3	57.3-96.1	0.65
	Cutters	3	72.5±8.0	57.1-86.8	0.70
IV - Very distinct	Sewers	2	81.3±6.6	64.5-98.0	0.56
	Cutters	1	84.1±3.9	70.5-97.4	0.60

*Possible scores 100 for each item.

Data displayed in Table 7 show that, in general, persons had light or moderate hypnotic trance manifestations and the scores were accordingly: for sewers 33.0±8.8 and 63,4±7.3, and for cutters - 40.3±8.3 and 57.8±8.6.

The pre-treatment to post-treatment changes in pain intensity and pain interference, stated by BPI scores are shown in Table 8.

**Table 8:** Pre- to post-treatment changes in composite pain intensity and composite paint interference, and 95% confidence interval (CI)

**Brief Pain Inventory composite scores**								
**Variable**	**Pre-treatment (mean±SD)**	**CI**	**Post-treatment (mean±SD)**	**CI**	**Follow up (after CH and SH treatment session)**			
					**After 3 months**	**CI**	**After 6 months**	**CI**
Pain intensity								
- sewers (n = 60)	11.1±2.8	8.3-13.9	9.6±1.5	7.2-12.0	8.4±1.8	6.3-10.5	9.2±2.2	6.8-11.5
- cutters (n = 30)	17.4±3.2	11.2-23.6	12.5±2.8	8.0-16.9	10.2±2.3	6.5-13.8	8.2±1.6	5.2-11.1
Pain interference								
- sewers (n = 60)	39.4±11.2	29.4-49.3	28.6±2.6	21.3-35.8	22.0±2.8	16.4-27.5	19.8±2.8	14.7-24.8
- cutters (n = 30)	25.9±9.8	16.6-35.1	16.8±2.1	10.7-22.8	12.7±1.9	8.1-17.2	10.6±2.3	6.4-13.5

As can be seen from Table 8, significant decrease of composite pain intensity and composite pain interference can be observed as the result of CH and SH therapy. It is evidenced by the pre-treatment data, which shows a relatively large number of the BPI score at the period in which only physical exercises were carried out. After the conclusion of CH and SH therapy, employees continued only with SH. Follow-up data show that a regular practice of SH was an effective means to decrease chronic pain after 3 months, which was testified by records in employees’ pain diaries. However, follow-up data after 6 months show that the effect of SH has slightly decreased as regards composite pain intensity indices for the representatives of both occupations. We believe this to be the result of not regular SH practice, as one can judge from the pain diaries.

Summing up the BPI scores regarding pain intensity and interference, descriptive statistics shows that after MH program decrease of the scores was found for employees with the length of service 8–15 years and more than 15 years.

#### MYO

**Figure 2:**
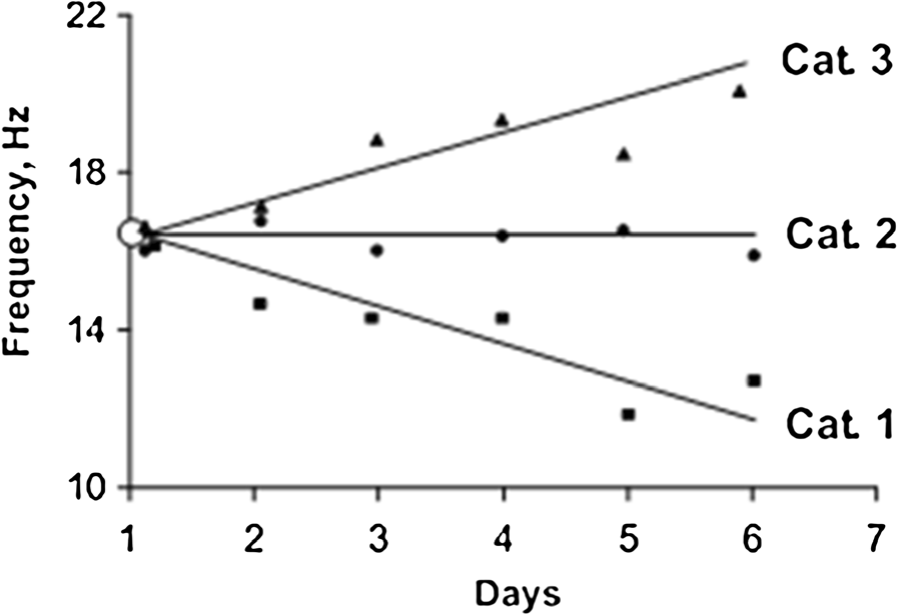
MYO Categories (example of the myotonometry testing data regression analysis of muscle frequency during consecutive 6 work days).

According to regression analysis of MYO data, the slope of the lines (trendline) reflects the condition of the muscles after one week work cycle [19]. Figure 2 shows the classification of the MYO data: Category I – subject is able to relax the muscle; Category II – muscle is able to adapt to the workload and to relax partly; Category III – muscle is not able to relax (muscle tone is increased which associate with muscles fatigue).

**Figure 3:**
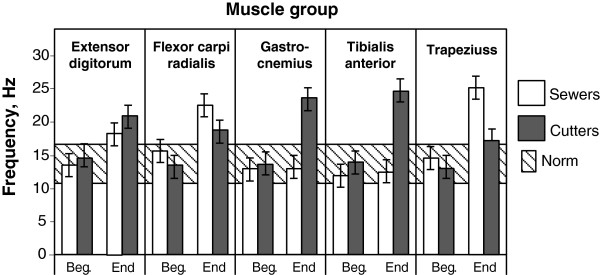
Illustration of frequency changing in separate muscle groups while performing the sewing and textile cutting at the beginning and at the end of the work week - for workers who are not able to adapt with the workload and whose muscle frequency exceeds the norm after the work week cycle.

MYO testing results for reference groups before and after rehabilitations are reflected in Tables 9 and 10. Testing results in Figure 3 show the frequencies of different muscles at the beginning and end of the work week for sewers and cutters, whose muscle frequency after the work week cycle exceeds the norm (11 up to 16 Hz, exist for each muscle individually). These frequencies show changes in the muscle tone for persons\ not able to adapt to workload. Therefore, the muscle fatigue is stated, and such workers can be referred to as belonging to III MYO category. These data were observed for both reference groups before MH program.

**Table 9:** **Percent of *sewers *(n = 60) and *cutters *(n = 30) with differences in their muscle tone (MYO Categories) before and after MH program, and Cohen’s Kappa (κ)**

**Before MH**		**After MH**	
**Category**	**κ**	**Category**	**κ**
Sewers:			
I - 0%	0.41	I - 10.0%	0.67
II - 70.0%	0.78	II - 83.3%	0.86
III - 30.0%	0.65	III - 6.7%	0.55
Cutters:			
I - 0%	0.76	I - 13.3%	0.67
II - 73.3%	0.88	II - 80.1%	0.86
III - 26.7%	0.65	III - 6.6%	0.55

**Table 10:** **Number of *sewers *(total n = 60, females n = 50, males n = 10) and *cutters *(total n = 30, females n = 20, males n = 10) with differences in their muscle tone (MYO Categories) before and after MH program depending on length of service in occupation**

**Length of service, years**	**Before MH**				**After MH**			
		**MYO Category**				**MYO Category**		
		**I**	**II**	**III**		**I**	**II**	**III**
A 0-7	Sewers (n = 20):				Sewers (n = 20):			
	*- females*	0	16	2	*- females*	4	14	0
	- *males*	0	2	0	- *males*	0	2	0
	Cutters (n = 12):				Cutters (n = 12):			
	*- females*	0	9	0	*- females*	0	9	0
	- *males*	0	3	0	- *males*	0	3	0
B 8-15	Sewers (n = 30):				Sewers (n = 30):			
	*- females*	0	19	6	*- females*	2	21	2
	- *males*	0	2	3	- *males*	0	4	1
	Cutters (n = 10):				Cutters (n = 10):			
	*- females*	0	3	2	*- females*	0	5	0
	- *males*	0	2	3	- *males*	0	4	1
C > 15	Sewers (n = 10):				Sewers (n = 10):			
	*- females*	0	3	4	*- females*	0	6	1
	- *males*	0	0	3	- *males*	0	3	0
	Cutters (n = 8):				Cutters (n = 8):			
	*- females*	0	3	3	*- females*	0	5	1
	- *males*	0	2	0	- *males*	0	2	0

Measurement results show that sewers’ muscle tone at the end of the working week has increased in shoulder region (*m. trapezius -* upper part) and wrist/hands (*m. extensor digitorum*; *m. flexor carpi radialis*). Cutters’ muscle tone at the end of the working week has increased in the most muscle groups in wrist/hands, in shoulder region, as well as in the legs: *m. extensor digitorum; m*. *tibialis anterior; m*. *gastrocnemius (caput mediale)*.

The percentage of the participants with differences in their muscle tone (MYO categories) before and after MH program is shown in Table 9.

It was also found that muscle tone and fatigue, as well as efficiency of MH, substantially depend on the length of service (in this study - in proportion with the age) rather than on gender, see Table 10. Workers’ physical fitness also is significant, and it characterizes the MYO categories at the end of MH program.

The results displayed in Table 10 show that after the MH program 6 female sewers, divided into service length reference groups A and B, with shoulder and neck muscles able to relax after one week work cycle, and thus meeting category I MYO (*κ* varies 0.67-0.86) were observed. It should be noted that after MH, for sewers with the length of service up to 7 years (group A) the increased muscle tone and fatigue (category III MYO) could not be observed in any of the examined muscle groups during one week work cycle (*κ* varies 0.55-0.75). After MH also the number of those sewers decreased (group B – from 6 to 2; group C – from 4 to 1), who formerly did fit into the category III MYO (inability to adapt to workload).

Analyzing efficiency of the MH program for male and female workers no differences could be found, because the muscle tone has decreased for both men and women. However, the difference could not be detected most likely due to the fact that very few male workers were represented in all reference groups.

**Figure 4:**
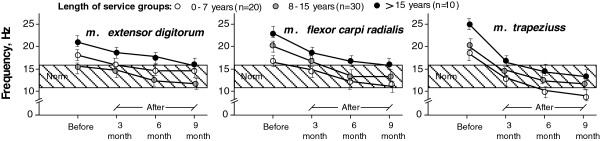
Development of the muscles tone among several sewers length of service groups before and after MH program (data show measurements at the end of working week).

To observe the changes of the muscle tone for workers from different service-length groups, during the whole MH program period the MYO measurements where done after every 3-months period (duration of the measurements was one workweek at the end of the 3rd month). The results for sewers reference group are shown in Figure 4.

**Figure 5:**
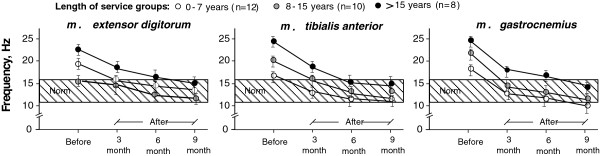
Development of the muscles tone among several cutters length of service groups before and after MH program (data show measurements at the end of working week).

What regards cutters, changes during a 9 month long period were analyzed for those muscle groups, which are the most characteristic for the workload when cutting textiles and for which the most frequent deviations from the norm could be stated (see Figure 3): *m. extensor digitorum*, *m*. *tibialis anterior,* and *m*. *gastrocnemius.* The results for cutters reference group are shown in Figure 5.

Data displayed in Figures 4 and 5 show that the effect of MH program on muscles tone can be observed in the 6th month, when, in general, all the examined muscle groups (especially *m. flexor carpi radialis* and *m. trapeziuss* for sewers; *m. extensor digitorum* and *m*. *tibialis anterior* - for cutters) reached parameters close to or within the norm (contraction frequency 11–17 Hz).

#### HRM

Heart rate monitoring was done to evaluate the work heaviness degree in terms of energy expenditure. For HRM, a 6-hour work period with rest breaks was chosen. Research results of HRM for sewing and cutting tasks are summed up in Table 11. Results of HRM are shown, taking into account average heart rate and energy expenditure of each person, standard deviation (SD).

**Table 11:** **Heart rate monitoring data for *sewers *and *cutters*: heart rate (HR), energy expenditure (E), Cohen’s kappa (κ), and work heaviness category (WHC)**

**Occupation**	**Mean HR ±SD, beats/min**	**Range HR, beats/min**	***κ***	**Mean E ± SD, kcal/min**	**WHC**
Sewers (females, n = 50)	79.5 ± 7.4	67-93	0.75	3.4 ± 0.5	Light work
Sewers (males, n = 10)	84.8 ± 3.9	66-93	0.78	3.6 ± 0.6	Light work
Cutters (females, n = 20)	120.3 ± 8.1	80-130	0.68	4.6 ± 0.5	Moderate work
Cutters (males, n = 10)	118.3 ± 9.5	75-120	0.68	4.7 ± 0.5	Light work

### Life quality assessment

**Figure 6:**
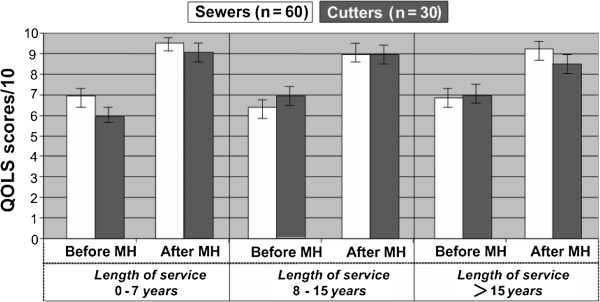
**Mean QOLS scores depending on length of service for sewers and cutters before and after the MH program (Cohen’s kappa - *κ *varies 0.76-0.88).**

Descriptive statistics based on rating scores for Quality of Life Scale before and after MR program is shown in Figure 6. Highest score possible is 100 (in conformity with 0 to 10 numeric rating scales for each 10 questionnaires), therefore, QOLS rating scores in Figure 6 are displayed as Total scores/10.

Analysis of the acquired QOLS scores allows concluding that after the completed MH program life quality notably improved for all reference groups. Especially this can be said about the youngest and eldest employees, for whom the life quality improvement could be stated regarding their social activities, activity at home, lack of plans for weekends, etc. (accordingly average score for the young - 6.5±0.5 before MH program and 9.25±0.5 after MH, for the elder - 6.9±0.3 before MH and 8.6±0.5 after MH program).

## Discussion

According to the aim of this work, the effectiveness of the MH program for sewers and cutters suffering from chronic pain in different bodily parts was assessed by analyzing musculoskeletal complaints, including chronic pain characteristics, features and muscle fatigue before and after MH program. Particular restrictions on total number of participants in MH program were determined.

There are persons who met the inclusion criteria, had a negative previous experience with medication therapy and had opportunity to participate in MH program with short follow-up period.

Analysis of the participants’ answers regarding pain in different parts of their body and its intensity reveals that chronic pain for sewers (see Table 3) basically is localized in the neck, shoulders and palms (accordingly 35.7%, 43.3% and 21.6% of the total number of respondents n = 300). For cutters, in their turn, chronic pain localizes not only in hands or neck/shoulders region, but in the leg muscles as well, due to prolonged work in a forced, awkward upright position (44.1% of the total number of respondents n = 50), see Table 4. It can be noted that using self-administered questionnaires symptoms subjectively may be either excessive or underestimated, but it didn’t influence the results and conclusions as research objective measurements (MYO and HRM) proved.

These statements were also proved using MYO measurements for the group of people who agreed to take part in an objective investigation (see Tables 9 and 10). MYO measurements show that the cause of chronic pain is not related to great workload, muscle fatigue and increase in muscle tone during the weekly work cycle. During the study it was stated that, before the MH program has been started, muscle tone in 70% of sewers (from n = 60) and 73% of cutters (from n = 30) met MYO category II, respectively, frequency of muscle contractions did not exceed norm. Consequently, their workload has adapted to the work speed and working conditions.

Therefore, presumably, muscle fatigue could not be considered to be the only reason of chronic pain. This complies also with the HRM data showing that dynamic workload is not very high neither for sewers nor cutters; as it can be seen from Table 11, energy expenditure in sewing and cutting tasks varies from E = 3.4 till 4.7 kcal/min. Therefore, sewers’ and cutters’ work heaviness can be referred to as light dynamic work (except female cutters’ work heaviness, which is estimated as moderately hard work).

Thus there emerged the question regarding the real cause of chronic pain. Analyzing the data acquired by MYO measurements and from the participants’ questionnaires, as well as from self-monitoring, it can be concluded that, in general, the employees suffered from psychosomatic chronic pain, caused by psycho-emotional and psychosocial factors, such as negative life events, psycho-traumatic interpersonal relationships at the workplace, mood disorders, and frustration related to work performance.

This was also proved during the research, because at the end of the MH program, including CH and SH, the impact of psychological factors significantly decreased, which was testified by the pain interference and life quality improvement (see Table 8 and Figure 6). These results can be explained with the inclusion of CH and SH in the MH program parallel to relaxation exercises. During these sessions the self-esteem of workers was raised, they mastered ability to relax, to overcome negative stress situations. Especially important this was for the employees with the length of service of 8-15 years, who – unlike the employees with a short or very long professional employment – suffered from a low self-esteem and poor ability to tolerate negative work and life stress. Thus it should be noted that inclusion of CH and SH in the MH program contributed to the reduction of chronic pain intensity, psychogenic tension and muscle fatigue. This was proved by objective measurements and survey of pain intensity and quality of life.

Comparison with previous studies shows that the injuries and muscle pain, caused by ergonomic risks, are common problems for clothing industry employees’ in several countries [32-34]. Therefore job conditions, found in this study, are comparable with earlier findings, and it is in accordance with other authors’ investigation results, where the prevalence of the persistent neck and shoulder disorders for sewing machine operators has been found to be increasing along with years of employment. The acquired data comply with the literature data on the importance of the usage of hypnotherapy and practice of self-hypnosis for operative individuals with chronic pain and manifestations of depression [35-38]. This is also in line with studies showing that causes of chronic pain can be linked with negative stress at workplace, emotional conflicts in the family, psychosocial problems, and depressive mood because the brain has a low natural anti-pain and anti-anxiety capacity [39]. Therefore, it has been suggested that there is a relationship between psychosocial factors and the persistence or recurrence of pain, and a multidisciplinary intervention, involving a workplace visit or more comprehensive occupational health care intervention, helps chronic or sub-acute patients to return to work faster [40].

Our interest in this examination was concentrated on the time period when the MH program (including remedial gymnastics) was efficient and allowed the selected muscle groups to relax and restore capacity to workload. It was found that during CH and SH sessions not only the decrease in psychogenic tension can be observed, but also the decrease in the tension caused by physical factors - several muscle groups were relaxed, which was testified by the diminishing muscle contraction frequency during the mentioned session and revealed using kinetic curves (see MYO data in Figures 4 and 5). The above mentioned objective ergonomic analysis, based on objective measurements, is confirmed by the subjective statements of workers (using questionnaires and interviews).

During our research low muscle frequencies were not examined, because the device does not allow measuring the deep muscles groups. Therefore it was not possible to prove that muscle pain, all sewers and cutters (total n = 90) complained of, were caused by physical muscle fatigue, for before the MH program MYO measurements showed an increased muscle fatigue (III MYO Category) only in 19 persons. Presumably, for most of the participants the intensity of pain and chronic sufferings relate to psychosocial risks, as was motivated in the discussion above. In further research it will be found out, which risk factors are decisive for the origin of pain. In this case the electromyography measurements are necessary.

The novelty of this research, regarding pain management for adult workers with chronic pain in Latvia, lies in the usage of CH treatment and SH during MH program, which could make some contribution directly to reducing effects of psycho-traumatic factors and work stress. Psycho-educational intervention, CH and SH in pain management with mind/body relaxation, pain-blocking imagery and desensitization of the psycho-traumatic events are very topical for patients. During CH treatment and SH workers with somatoform pain disorders are taught to concentrate on the images, promoting muscle relaxation and is guided to the healing imagery through catharsis and ego-strengthening. This complies with findings about hypnosis and self-hypnosis treatment for patients with back pain [40,41].

According to the data acquired from QOLS questionnaires, before MH program all sewers (n = 60) and cutters (n = 30) in all service length groups with chronic pain in NSAH and legs marked a comparatively low quality of life (limited social activities, activity at home, lack of plans for weekends, etc.). After MH the quality of life substantially increased, and all persons, who took part in MH, started actively participating in social activities, such as family life, outdoor activities, and active working hours.

## Conclusion

The results, acquired during the research, convince of efficiency of medical hypnotherapy, including cognitive hypnotherapy and self-hypnosis, in the treatment of employees suffering from chronic pain in their neck/shoulders region, arms and legs. For all workers (sewers and cutters), involved in the objective investigation, the intensity of chronic pain significantly decreased in different parts of their body, as did manifestations of depression, they became physically more active, muscle fatigue lessened and other life quality indices improved. Use of cognitive hypnotherapy and self-hypnosis sessions in medical hypnotherapy program allows workers to decrease not only psychogenic tension, but also the tension caused by physical workload. It is proved by myotonometric measurements and heart rate monitoring. Therefore these measurements are suitable for objective determination of the state, tone and fatigue of muscle groups, as well as employee’s workload.

## Abbreviations

MH: Medical hypnotherapy; WRMDs: Work-related musculoskeletal disorders; NSAH: Neck shoulders arms and hands; NMQ: Nordic musculoskeletal questionnaire; BPI: Brief pain inventory; QOLS: Quality of life scale; STS: Sunnen trance scale; MYO: Myotonometry; HRM: Heart rate monitoring; PPR: Prevalence proportion ratio; CI: Confidence Interval; CH: Cognitive hypnotherapy; SH: Self-hypnosis.

## Competing interests

The authors declare that they have no competing interests.

## Authors’ contributions

All authors made substantial contributions to the study conception and design of the study, detailed literature search, acquisition of the data, its statistical analyses and final interpretation. All authors approved the final version for submission.

## Authors’ information

Zenija Roja, MD, PhD, as. professor, University of Latvia, Center for Ergonomics Research, Faculty of Chemistry, K.Valdemara str. 48, LV-1013, Riga, Latvia; President of Latvian Ergonomics Society. Phone: +371 29563591; e-mail: zenija.roja@lu.lv. Valdis Kalkis, PhD, Dr.habil.chem, professor, University of Latvia, Ergonomics Research Centre, Faculty of Chemistry, K.Valdemara str. 48, LV-1013, Riga, Latvia; Board member of Latvian Ergonomics Society. Phone: +371 29198476; e-mail: valdis.kalkis@lu.lv. Inara Roja, MD, PhD, clinical neurologist, hypnotherapist, Out-patient Department of the Riga 1st Hospital, Riga, Bruninieku str. 5, LV-1001, Riga, Latvia; President of Latvian Medical Society of Hypnotherapists; Phone: +371 29822588; e-mail: inara.roja@gmail.com. Henrijs Kalkis, PhD, University of Latvia, Faculty of Economics and Management, Aspazijas Boulevard 5, Riga, LV-1050. Phone, Riga, Latvia; Board member of Latvian Ergonomics Society; Phone: +371 29739399; e-mail: henrijs.kalkis@lu.lv.
